# Characterization of antigenic variants of hepatitis C virus in immune evasion

**DOI:** 10.1186/1743-422X-8-377

**Published:** 2011-07-29

**Authors:** Jane H Wang, Matthew J Pianko, Xiaogang Ke, Alex Herskovic, Ronald Hershow, Scott J Cotler, Weijin Chen, Zheng W Chen, Lijun Rong

**Affiliations:** 1 Section of Hepatology, Department of Medicine, University of Illinois at Chicago, Illinois, USA; 2 Division of Gastroenterology, Hepatology and Nutrition, Department of Pediatrics, the Feinberg School of Medicine, Northwestern University, Chicago, Illinois, USA; 3 Department of Microbiology and Immunology, University of Illinois at Chicago, Illinois, USA; 4 Division of Epidemiology and Biostatistics, School of Public Health, University of Illinois at Chicago, Illinois, USA; 5 Changchun Institute of Biological Products, China National Biotec Group Int. Changchun, China

## Abstract

**Background:**

Antigenic variation is an effective way by which viruses evade host immune defense leading to viral persistence. Little is known about the inhibitory mechanisms of viral variants on CD4 T cell functions.

**Results:**

Using sythetic peptides of a HLA-DRB1*15-restricted CD4 epitope derived from the non-structural (NS) 3 protein of hepatitis C virus (HCV) and its antigenic variants and the peripheral blood mononuclear cells (PBMC) from six HLA-DRB1*15-positive patients chronically infected with HCV and 3 healthy subjects, the *in vitro *immune responses and the phenotypes of CD4^+^CD25^+ ^cells of chronic HCV infection were investigated. The variants resulting from single or double amino acid substitutions at the center of the core region of the Th1 peptide not only induce failed T cell activation but also simultaneously up-regulate inhibitory IL-10, CD25^-^TGF-β^+ ^Th3 and CD4^+^IL-10^+ ^Tr1 cells. In contrast, other variants promote differentiation of CD25^+^TGF-β^+ ^Th3 suppressors that attenuate T cell proliferation.

**Conclusions:**

Naturally occuring HCV antigenic mutants of a CD4 epitope can shift a protective peripheral Th1 immune response into an inhibitory Th3 and/or Tr1 response. The modulation of antigenic variants on CD4 response is efficient and extensive, and is likely critical in viral persistence in HCV infection.

## Background

It is estimated that hepatitis C virus (HCV) infects at least four million Americans and more than 120 million individuals globally [1]. Each year in the United States an additional 30,000 new infections occur; and over 70% of patients develop chronic infection leading to end stage of liver diseases and in many cases, death [2,3]. It is clear that an effective anti-viral cellular immune response is critical for viral clearance and prevention of chronic HCV infection [4-14]. Recent studies also reported the importance of innate immunity and interferon lambda in the control of HCV infection [15,16]. However, the mechanisms by which HCV evades host immune defenses and establishes persistent infection remain to be elucidated. It is known that antigenic variation is an effective way by which viruses avoid immune recognition and may play a critical role in the development of viral persistence in infections with HCV [17], human immunodeficiency virus (HIV) [18], influenza virus and other viral diseases [19].

RNA viruses evolve at very rapid rates, which is due to the lack of a proofreading capacity of the RNA-dependent RNA polymerase [20], a characteristic that is recognized as the basis of their adaptability. Consistent with this, HCV in an infected patient consists of quasi-species that have distinct but closely related RNA sequences. Current hypotheses relate the tendency of HCV infection to persist to the existence of virus quasi-species and emergence of antigenic variants driven by immune selection [21-24]. Meaningful antigenic variation results from mutations in protein regions targeted by antibody and T cells [25]. There is evidence that naturally occurring variants of CD8 epitopes act as T-cell receptor antagonists for antiviral cytotoxic T cell response [17,18,26,27]. A recent study showed that HCV accumulates clustered mutations within an immune-dominant epitope, viral protein RdRp which is bound by HLA-B27 molecule. HCV escapes CD8 T-cell immune response in HLA-B27+ patients through mutating in the RdRp fragment [28]. Large viral diversity during the acute phase of HCV infection has been associated with the progression to chronic infection, whereas recovery from infection has been associated with relatively smaller viral diversity [19].

T-helper (Th) CD4 cells, the other key component of adaptive immunity, also play a major role in host defense against viruses and intracellular microbes [29]. Clonal expansion and maintenance of CD8 activity depend upon specific Th1 cells [30]. A protective Th1 response, characterized by Th1 cytokines such as interferon (IFN)-γ is essential for viral clearance. It was reported that the absence of an adequte CD4 response is associated with incomplete control of HCV replication by memory CD8 cells and failure to resolve HCV infection [29]. A strong HCV-specific Th1 response was observed in patients who resolved acute HCV infection, whereas patients who were unable to mount a CD4 response developed chronic infection [29,31]. The mechanisms by which HCV escapes CD4 responses are remained unclear. Understanding how HCV escapes an initial CD4 response should provide insight into the pathogenesis of chronic HCV infection.

Unlike HIV, HCV does not cause a systemic ablation of the immune system but rather is associated with a form of specific tolerance such that immune responses to HCV are blunted and are unable to eliminate the viruses [8]. Regulatory T (Treg) cells are central to the control of immune reactivity [32] and are important in organ transplantation [33]. Antigen inducible Tregs act to suppress inflammation and prevent tissue and organ injury during responses to infection, mainly by secretion of IL-10 (Tr1) and TGF-β (Th3)[32]. Studies by others have shown that CD4^+^CD25^+ ^Tregs and IL-10-producing Tr1 cells modulate the proliferation of HCV-specific CD4 and CD8 cells in patients infected with HCV for 20~30 years [34-37]. These data may reflect a well established immune tolerance after lengthy HCV infection. Yet, it is not clear what mechanisms induce expression of Tregs, the precise phenotypes of Tregs that are involved in developing immune suppression and what role HCV antigenic variants play in immune evasion during the early course of infection.

Our previous studies using peripheral blood mononuclear cells (PBMCs) from a patient infected with HCV for about 2.5-4 years identified a Th1 epitope and its variants derived from the non-structural (NS) 3 protein of HCV [38]. We also found that variants of the Th1 epitope can effectively suppress host polyclonal peripheral T cell proliferation, and shift the cytokine secretion patterns from one characteristic of a Th1 antiviral responses to a Th2 form [39,40]. In the present study we show how such CD4 variants act as antigenic variants that modulate T-cell function through multiple mechanisms in early HCV infection.

## Materials and methods

### Patient samples

All samples were obtained with informed consent and approval of the local Institutional Review Board (IRB). Peripheral blood samples were collected from a patient (B3019) with chronic hepatitis at about 18~28 months and four years after infection. PBMCs were isolated as described previously [40]. The HLA-class II alleles of this patients are HLA-DR*1501 and DR*0701. PBMCs from five HLA-DRB1*15-positive patients and three healthy DRB1*15-positive individuals were also used for *in vitro *detection of CD4^+^CD25^+ ^Tregs. Fresh PBMCs from additional six patients infected with HCV for about 2~5 years and six healthy individuals were also isolated and used for determination of *in vivo *levels of CD4^+^CD25^+ ^Tregs. The detailed information of HCV-infected patients is listed (see Additional File 1, Table S1). The presence of HCV-specific antibodies in patients' sera and HCV genotypes were determined as described previously [40].

### Peptides

**Figure 1:**
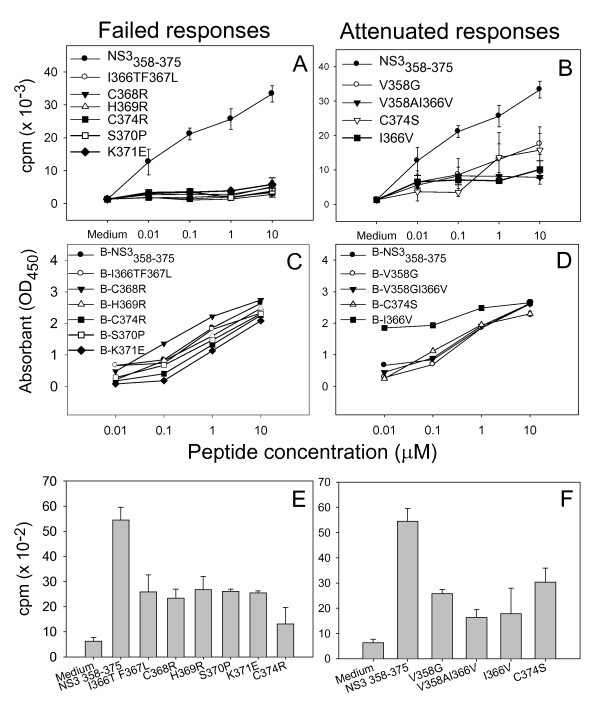
Proliferation in response to peptides of wild-type NS3_358-375 _and its variants and binding affinity of those peptides to HLA-DRB1*1501 molecules. **A and B**: PBMC from a HCV-infected patient B3019 were cultured with wild type NS3_358-375 _and variant peptides at the given concentrations, to six days, pulsed overnight with 1.0 μCi/well tritiated thymidine. Radioactive label incorporation (cpm) was measured. PBMC with tissue culture medium alone was used as a negative control. Experiments were performed in triplicate. A representative of five experiments is shown. **C and D: **Binding of biotinylated peptides to HLA-DRB1*1501 molecules were assessed using an antigen-capture ELISA. Overnight binding affinities of variant peptides were compared to wild type peptide NS3_358-375_. A representative of three experiments is shown. **E and F: **PBMCs were pre-pulsed with 0.1 μM variant peptides for three hours, then re-challenged with wild type NS3_358-375 _at a concentration of 10 μM. Rest of the experiment was as same as mentioned in A. A representative of five experiments is shown.

A CD4 epitope derived from the non-structural (NS) 3 protein of HCV and its antigenic variants were identified as previously described [40,41]. Peptides corresponding to wild type and variant sequences of NS3_358-375 _were synthesized [40,41]. The sequences of NS3_358-375 _wild type and variant peptides are listed in Table 1. Variant peptides were also pooled into two groups, VP1 and VP2 (Figure 1D), according to their proliferation responses for further experiments.

**Table 1:** Synthetic peptides of NS3_358-37__5 _and its variants

Peptide name		Amino acid sequence	Time of isolation*
Wild type	NS3_358-375_	VIKGGRHLIFCHSKKKCD	
VP1**	I366T F367L	--------TL--------	1.0
	C368R	----------R-------	1.0
	H369R	-----------R------	1.0
	S370P	------------P-----	1.0, 2.4
	K371E	-------------E----	1.0
	C374R	----------------R-	2.4
VP2	V358G	G-----------------	1.0
	V358A I366V	A-------V---------	1.0
	I366V	--------V---------	2.4
	C374S	----------------S-	2.4

*: years after infection. **: VP means variant pool.

Note: the core region of NS3_358-37 _encompasses amino acids 364-373.

### Proliferation

Proliferation with each peptide was performed as described previously [39].

T cell proliferation with variant pools of VP1 and VP2 was measured using thymidine incorporation assay as described previously [39]. In this assay, PBMC were re-suspended at a concentration of 10^6^/ml in RPMI 1640 tissue culture medium containing 25 mM HEPES, 2.0 mM L-glutamine, 100 U/ml penicillin, 100 μg/ml streptomycin, and 10% pooled human plasma (PHP). Two 100 μl aliquots of PBMC mentioned above were cultured with either wild peptide NS3_358-375 _(5 μM) or variant-peptide pool VP1 and VP2 (1 μM/each peptide) for three hours in 96-well round bottom plates. Then 15 μg/ml of each antibody to human IL-10 (Pierce Endogen, Rockford, IL, USA), CD25, and TGF-β1 (BD biosciences, San Diego, CA, USA) were added. Parallel cultures containing either wild type peptide, or variant-peptide pools without antibody, or PBMC with medium alone were used as controls. Cultures were incubated at 37°C in 5% humidified CO_2. _At day four, cells were pulsed overnight with 1.0 μCi/well tritiated thymidine. Radioactive label incorporation (cpm) was measured at day five. PBMC stimulated with wild type NS3_358-375 _alone and medium without antigen were used as positive and negative controls respectively. Results were represented as mean ± SEM cpm of duplicate cultures.

### Anergy assay

To measure induction of unresponsiveness by variant NS3 peptides, PBMCs were pulsed with 0.1 of each variant peptide for 3 hours at 37°C. Free peptide was washed out and 100 μl aliquots of the cells at 1 × 10^6^/ml were added to wells containing wild type NS3_358-375 _peptide at 10 μM. After 65 hours, cultures were assessed for proliferation by thymidine incorporation. PBMC stimulated with wild type NS3_358-375 _alone and medium without antigen were used as positive and negative controls respectively.

### Peptide binding

An assay for *in vitro *peptide binding was developed as previously described to determine relative HLA binding levels of wild type and variant peptides [39]. Binding affinities of biotinylated-variant peptides to HLA-DRB1*1501 molecules were compared to that of wild type peptide.

### Cytokine ELISA

ELISAs for IL-2, IL-10, IFN-γ and TGF-β were performed using specific ELISA kits according to manufacturer's instruction (BD Pharmingen) as described previously [39].

### Intracellular cytokine staining and flow cytometry

#### Antibodies and reagents

The following antibodies from BD Biosciences were used for cell separation and staining: purified mAb for human CD4, FITC-conjugated IFN-γ, IL-2, PE-conjugated IL-10 and CD152 (CTLA-4), APC-conjugated CD25, biotinylated-TGF-β1, PECy7-conjugated-streptavidin, FITC-conjugated mouse IgG1, PE-conjugated mouse IgG 2a and rat IgG 2a, biotin-conjugated rat IgG2a. FITC-conjugated *Foxp3 *(eBiosciences) and ECD-conjugated CD25 and mouse IgG2a (Beckman Coulter, Miami, FL, USA) were also used.

### Separation of CD4^+ ^cells

PBMCs were re-suspended at a concentration of 1 × 10^6 ^/ml in RPMI 1640 tissue culture medium as mentioned above in the description of the proliferation assay. 1 ml aliquots of PBMC were added to a 24-well tissue culture plate containing each wild type NS3_358-375 _or variant pool, and medium alone. The concentrations of peptides are 5 μM for NS3_358-375 _and 1 μM for each of variant peptides. After culturing for 66 hours, the PBMCs were treated with anti-human CD4 antibodies and magnetic micro-beads (Miltenyi Biotec, Auburn, CA) for positive selection of CD4 cells using a Magnetic Cell Separator (MACS, Miltenyi Biotec). The purity of CD4^+ ^cells obtained was 99.3% determined by Flow Cytometry.

### Cellular staining and flow cytometry assay

The purified CD4^+ ^cells were treated with 0.5 ml of fix/permeabilizing solution (BD Pharmingen) for 10 minutes. After washing, the appropriate volume of specific fluorescent-labeled anti-CD25-ECD, anti-IFN-γ-FITC, anti-IL-10-PE, anti-TGF-β1-biotin and streptavidin-CyChrome were added to each of the tubes. One additional tube of CD4^+ ^cells were incubated with *Foxp3*-FITC, CD152 (CTLA-4)-PE and CD25-ECD. A tube of pooled CD4^+ ^cells from each culture was stained with isotype antibodies equivalent to each of the antibodies described above. In addition, four tubes of pooled CD4^+ ^cells were stained with each of the above antibodies alone as single-color controls which is necessary for color compensation. All tubes were incubated for 30 minutes in dark at room temperature. After washing, cells were re-suspended in 300 μl of wash buffer (BD Pharmingen), and a four color or three-color flow cytometry assay was performed within 24 hours at the Flow Cytometry Service Laboratory of the University of Illinois at Chicago. A total 1 × 10^5 ^cells were counted to determine the ratio of CD25^+^, CD25^- ^and cytokine producing cells of each CD4^+ ^population.

### Statistical analysis

The phenotypes of CD4^+^CD25^+ ^Tregs of the six HLA-DRB1*15-positive patients and three healthy subjects were summarized in Table 2. The *in vivo *Treg levels and phenotypes of six other patients and six healthy subjects are shown in Table 3. Data are expressed as medians and ranges (Table 2). The p-values are from a Wilcoxon non-parametric t-test.

**Table 2:** Phenotypes of CD4+CD25+ cells induced by HCV variants

Median (range) (%)			
CD4+ cells	HCV- (n = 3)	HCV+ (n = 6)	p value*
Medium			
CD25+	8.31 (6.0 - 11)	23.4 (19 - 28)	0.03
TGFβ+	5.28 (3.5 - 9.0)	56.0 (46 - 61)	0.01
IL-10+	3.22 (2.5 - 3.5)	7.54 (6.0 - 17)	0.04
TGFβ+ IL-10+	0.67 (2.0 - 5.5)	7.85 (5.0 - 14)	0.04
NS3_358-375_			
CD25+	10.1 (10 - 14)	21.6 (18 - 27)	0.02
TGFβ+	5.04 (2.0 - 8.5)	55.4 (37 - 59)	0.01
IL-10+	2.95 (2.6 - 3.2)	6.51 (3.0 - 11)	0.03
TGFβ+ IL-10+	0.67 (0.5 - 2.5)	7.85 (5.0 - 12)	0.002
VP1			
CD25+	10.2 (7.0 - 13)	29.9 (21 - 48)	0.01
TGFβ+	6.18 (2.0 - 14)	57.1 (42 - 61)	0.06
IL-10+	5.77 (3.5 - 7.5)	6.23 (5.0 - 13)	0.33
TGFβ+ IL-10+	7.84 (2.0 - 6.5)	8.65 (5.0 - 15)	0.21
VP2			
CD25+	8.60 (5.0 - 13)	29.3 (23 - 51)	0.02
TGFβ+	7.04 (3.0 - 13)	58.8 (44 - 67)	0.04
IL-10+	6.57 (6.0 - 8.5)	7.73 (5.5 - 12)	0.36
TGFβ+ IL-10+	3.05 (2.5 - 7.0)	9.36 (5.0 - 17)	0.07

*: Wilcoxon non-parametric *t*-test

All subjects are positive for HLA-DRB1*15.

**Table 3:** Phenotypes of CD4+ regulatory cells in early HCV infection

Median (range) (%)			
CD4+ cells	HCV- (n = 3)	HCV+ (n = 6)	p value*
CD25+	9.50 (9.1 - 10)	20.9 (17 - 34)	0.02
TGFβ+	2.89 (1.3 - 8.4)	30.0 (25 - 37)	0.02
IL-10+	3.02 (1.6 - 5.1)	5.64 (2.0 - 16)	0.04
IFNγ+	5.04 (1.6 - 9.8)	5.14 (2.9 - 8.7)	0.81
TGFβ+ IL-10+	1.33 (0.3 - 3.4)	5.03 (1.4 - 9.3)	0.05
TGFβ+ IFNγ+	1.52 (0.7 - 5.0)	2.01 (0.9 - 6.8)	0.54
CD25-	56.0 (52 - 58)	51.3 (48 - 54)	0.07
TGFβ+	1.43 (0.7 - 4.2)	26.5 (20 - 33)	0.02
IL-10+	0.42 (NA**)	0.92 (0.5 - 6.8)	NA
IFNγ+	2.15 (0.9 - 3.2)	2.31 (0.4 - 7.3)	0.81
TGFβ+ IL-10+	0.08 (NA)	0.40 (0.2 - 2.5)	NA
TGFβ+ IFNγ+	0.07 (0.05 - 1.1)	0.70 (0.03 - 1.1)	0.2

*: Wilcoxon non-parametric *t*-test.

**: number of control subjects is less than 2.

## Results

### Lack of response to HCV antigenic variants

Our previous studies identified a CD4 epitope derived from the NS3 protein of HCV, NS3_358-375 _[38]. This epitope is human leukocyte antigen DRB1*15 (HLA-DRB1*15)-specific, since it binds to HLA-DRB1*1501 well, but not to the other class II allele of the patient, HLA-DRB1*07 [39]. Antigenic variants of the Th1 epitope were identified by RT-PCR, cloning and sequencing [41]. Peptides of wild type NS3_358-375 _and its variants (single or double amino acid substitutions) were synthesized and used in the current study (Table 1).

NS3_358-375 _variant peptides were compared to wild type peptide in T-cell proliferation assays with PBMCs collected from patient B3019 at about four years after HCV infection. Proliferation was abolished in the presence of variant peptides I366TF367L, C368R, H369R, S370P, K371E and C374R (Figure 1A: failed responses), and attenuated by variants V358G, V358AI366V, I366V and C374S (Figure 1B: attenuated responses). These two groups comprise 55% of the total number of the variants tested. Among the remaining variants, 39% (7/18) stimulated T cell proliferation at the levels compatible to that of wild type peptide; and only one variant, I366V (1/18, 6%), stimulated stronger T cell proliferation compared to wild type peptide (data not shown). Flow cytometry analysis showed that greater than 95% of peripheral proliferating cells stimulated by wild type NS3_358-375 _were CD4^+^. These data are consistent with our previous studies of three DRB1*15^+ ^patients infected with HCV [39]. Compared to our previous study (36), more variants showed inhibitory effect on T cell proliferation as shown in Figure 1A and 1B. The data reflects the progress of immune inhibition during the evolution of HCV infection.

When PBMCs were pre-pulsed with 0.1 μM of each variant peptide for three hours, then re-challenged with wild type NS3_358-375 _at a concentration of 10 μM, the cells showed greatly reduced responsiveness characteristic of anergy (Figure 1E-F). Even the variant peptides stimulating T cell proliferation at compatible levels as wild type peptide induced anergy (data not shown).

To determine whether failure to stimulate proliferation might be caused by a failure to bind the HLA-DR15 molecules, binding of biotinylated variant peptides to DRB1*15 molecules were compared to wild type NS3_358-375 _in an antigen capture ELISA. As shown in Figure 1C and 1D, dose-dependent binding was observed for each variant at levels comparable to or in excess of wild type peptide. These results demonstrate that the failure to stimulate T-cell proliferation cannot be explained as a failure to bind HLA molecules on the surface of antigen presenting cells (APC).

### The suppression by antigenic variants is mediated by IL-10 or TGF-β^+ ^cells

We reasoned that the effect of CD4 variants on immune responses *in vivo *should be the combined effect of all or some of the variants since viral variants do not exist in isolation *in vivo*. Therefore, we pooled the variant peptides into two groups, VP1 and VP2 (Table 1), according to their proliferation responses (Figure 1A and 1B) to better reflect the effect of CD4 variants on T cell responses *in vivo*.

**Figure 2:**
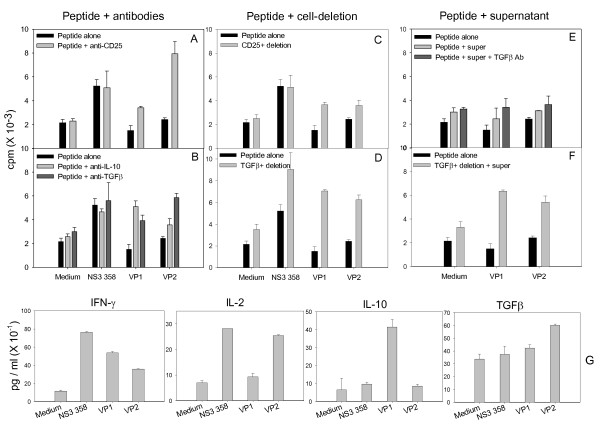
**Secreted IL-10, CD25^+ ^and TGF-β-bound cells were involved in the inhibition by HCV variants**. **A-B**: PBMCs were cultured with peptide NS3_358-375 _(5 μM), variant-peptide pool VP1, or VP2 (1 μM/each peptide) for three hours, then 15 μg/ml of each antibody to human IL-10, CD25 and TGF-β were added. Parallel cultures with either NS3_358-375 _or VP1 and VP2 without antibody, and PBMC with medium alone were used as controls. At day four, cells were pulsed overnight with 1.0 μCi/well tritiated thymidine. Radioactive label incorporation (cpm) was measured at day five. **C-D**. CD25^+ ^or TGF-β-bound cells were deleted from PBMCs using antibodies to CD25^+ ^or TGF-β and magnetic beads. The remaining cells were cultured with either NS3_358-375 _or VP1 and VP2. Proliferation measurement and controls were the same as described in A-B. **E**: 50 μl of a supernatant pool alone or the pool with 15 μg/ml TGF-β antibody was added into each culture of PBMC with VP1 or VP2. The supernatant pool consisted of each culture of VP1 and VP2 described in A. **F**. PBMCs without TGF-β-bound cells were cultured with VP1 or VP2 and 50 μl of the supernatant pool. All experiments were repeated twice, a single representative experiment is shown **G**. Levels of IFN-γ, IL-10 and TGF-β induced by peptide NS3_358-375_, VP1 and VP2 were determined by cytokine-specific ELISA. Each 100 μl of supernatant from the cultures described in A was used for ELISA analysis of IFN-γ, IL-10 and TGF-β. Experiments were performed in duplicate. A single representative of five experiments is shown.

We evaluated whether immunoregulatory cytokines such as IL-10 and TGF-β or CD25^+ ^regulatory cells play a role in the immune inhibition induced by VP1 and VP2. PBMCs were cultured with wild type peptide NS3_358-375 _and variant-peptide pools of VP1 and VP2 in the presence or absence of antibodies to human IL-10, TGF-β1 and CD25. As shown in Figure 2, the inhibition on T cell proliferation induced by VP1 was restored to the level of wild type peptide by anti-IL-10 (Figure 2B) and was partially restored by antibodies to CD25 and TGF-β (Figure 2A and 2B). These results demonstrate that IL-10 elaboration alone was sufficient to suppress T cell proliferation. In contrast, the inhibition induced by VP2 was completely blocked by antibodies to CD25 and TGF-β and was partially reversed by IL-10 antibody (Figure 2A and 2B), indicating that CD25^+ ^and TGF-β are critical in the inhibition by VP2. Consistent with these results, VP1 induced higher levels of IL-10 than wild type peptide and VP2, and VP2 induced lower levels of IL-10 and higher levels of TGF-β (Figure 2G). VP2 also induced similar level of IL-2 compared to wild type peptide, and both VP1 and VP2 induced lower levels of IFN-γ than that of wild type peptide (Figure 2G), suggesting modulation of a Th1 response by both VP1 and VP2 peptides.

To further examine whether CD25^+ ^or TGF-β^+ ^Tregs were directly involved in the inhibitory effect of HCV variants, CD25^+ ^or TGF-β^+ ^cells were deleted from PBMCs using antibodies to CD25 and TGF-β as well as magnetic beads. TGF-β exists either inside of TGF-β-secreting cells or bound to the surface of cells expressing TGF-β receptors. It is possible that TGF-β-secreting cells express TGF-β receptors as well. Thus the deletion of TGF-β^+ ^cells could be deleted both the TGF-β receptor expressing cells and the TGF-β-secreting cells. Those deleted cells are called TGF-β^+ ^cells here after. The remaining CD25^- ^or TGF-β^- ^cells were cultured with either wild type peptide or VP1 and VP2, and the proliferation was measured at day five. As shown in Figure 2D, the suppressive effect on proliferation by VP1 and VP2 was substantially reduced by deletion of TGF-β^+ ^cells indicating a crucial role of TGF-β^+ ^Th3 cells in the immune modulation induced by antigenic variants. An example of CD4^+ ^CD25^+ ^cells induced by VP1 cultured with anti-TGF-β antibodies or with deletion of TGF-β^+ ^cells is shown (see Additional File 2, Figure S1). Deletion of CD25^+ ^cells only partially reverted the suppressions by both VP1 and VP2 suggesting the possibility of the involvement of CD25^- ^suppressors in the reaction.

In order to evaluate whether soluble TGF-β or TGF-β-producing Th3 cells played a critical role in the inhibition by antigenic variants, we co-cultured PBMC and VP1 or VP2 with a supernatant pool consisting of supernatant from each culture of VP1 or VP2 with PBMC. The supernatant pool contained TGF-β at a concentration of 690 pg/ml determined by ELISA as previously described [40]. TGF-β in the supernatant pool was neutralized by TGF-β antibody, and then the supernatant was added into each culture containing VP1 or VP2. Cultures with peptide alone or peptide plus supernatant pool without neutralization of TGF-β were used as controls. As shown in Figure 2E and 2F, neutralization of TGF-β in the supernatant pool did not restore responses to VP1 and VP2. However deletion of TGF-β^+ ^cells did restore the responses even in the presence of the supernatant pool containing TGF-β. These findings suggest that TGF-β^+ ^Th3 cells rather than soluble TGF-β play a key role in a cell-contact mechanism in the immune inhibition caused by antigenic variants during early HCV infection. These observations also suggest that the mechanisms of immune modulation induced by VP1 and VP2 are distinct: it is mediated by IL-10 and CD25^-^TGF-β^+ ^cells for VP1 whereas by CD25^+^TGF-β^+ ^cells for VP2. Taken together, these findings indicate that HCV variants actively up-regulate a suppressive response rather than simply down-regulate a protective Th1 response.

### Phenotypes of pathogen-inducible Tregs driven by antigenic variants

**Figure 3:**
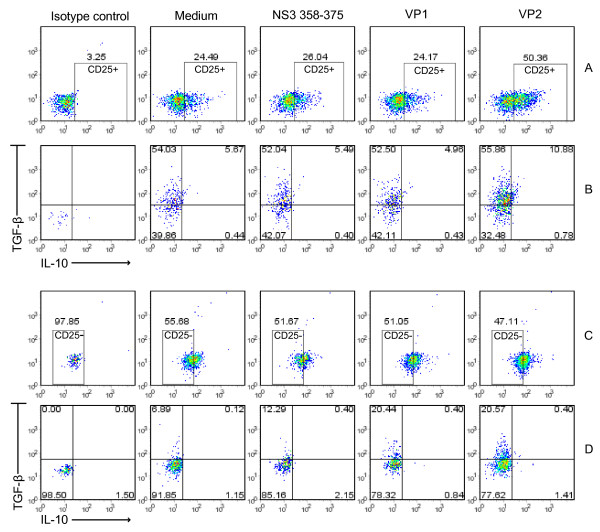
**Treg phenotypes induced by peptide NS3_358-375 _and variant pools VP1 and VP2**. Cell culture adn purification of CD4^+ ^cells and controls were the same as described in Figure 2. Intracellular staining and four-color flow cytometry using fluorescent-labeled antibodies to human CD25, IFN-γ, TGF-β and IL-10 were performed with purified CD4^+ ^cells. A pool from each test was used as an isotype control. **A**: Percentages of CD25^+ ^gated CD4^+ ^cells. Note that the percentage of CD25^+ ^cells stimulated by VP2 is significantly higher. **B**: Percentages of Th3 and Tr1 cells. Note that 58~67% of the CD25^+ ^cells were TGF-β^+ ^(sum of the upper two quadrants). A majority of the CD25^+^IL-10^+ ^cells were also TGF-β^+ ^(the upper right quadrant). **C: **Percentages of CD25^- ^gated CD4^+ ^cells. **D**: Percentages of Th3 and Tr1 cells. Note that the percentages of CD25^-^TGF-β^+ ^cells stimulated by VP1 and VP2 are significantly higher than stimulation with wild type NS3_358-375 _and medium alone. Two representatives of eight experiments are shown.

We sought to determine the Treg phenotypes responsible for IL-10 or TGF-β-mediated immune modulation by antigenic variants in early HCV infection. We detected CD4^+^CD25^+ ^and CD4^+^CD25^- ^cells and their cytokine secretion of IFN-γ, IL-10 and TGF-β by intracellular staining and flow cytometry. As shown in Figure 3A, VP2 induced significantly higher ratios of CD4^+^CD25^+ ^cells (50.4%) than wild-type peptide (26.0%) and VP1 (24.2%). A large proportion (57.5%~66.7%) of the CD4^+^CD25^+ ^cells were TGF-β^+ ^(Figure 3B, sum of the upper two quadrants), providing supporting evidence for the involvement of CD25^+^TGF-β^+ ^Th3 cells in the immune transition caused by VP2. As controls, the PBMCs from a HLA-DRB1*15^+ ^and HCV-negative healthy individual were cultured with wild type peptide NS3_358-375_, concanavalin A (ConA) (positive control) and medium alone. The ratios of CD25^+ ^and CD25^+^TGF-β^+ ^cells were ranged 13.9~17.8% and 5.04~6.16%, respectively(see Additional File 3, Figure S2A-B). We also observed CD25^+^IL-10^+ ^Tr1 cells induced by HCV peptide and its variants at a frequency of about 5~12% (Figure 3B, the sum of the lower and upper right quadrant), compared to normal controls (1~3%, Figure S2B). Over 90% of such Tr1 cells from the HCV infected patient were also TGF-β^+ ^(Figure 3B), whereas less than 50% of CD25^+^IL-10^+ ^cells from the normal control were TGF-β^+ ^(Figure S2B). The percentages of IFN-γ^+ ^cells (including both CD25^+ ^and CD25^-^) were less than 1% for the patient and ranged 1~4% for the normal control suggesting an attenuated Th1 response in HCV infection.

The fact that deletion of CD25^+ ^cells only partially reverted the suppressions by both VP1 and VP2 suggested the possibility of the involvement of CD25^- ^suppressors in the reaction. When CD4^+^CD25^- ^cells were analyzed, higher percentages of CD25^-^TGF-β^+ ^cells were observed for VP1 (20.8%) and VP2 (21.0%) than wild type NS3_358-375 _(12.7%, Figure 3D), suggesting such CD25^- ^Th3 cells could be the major source of TGF-β^+ ^cells that played an important role in the inhibition induced by VP1. The percentage of CD25^- ^Th3 cells from the normal control ranged 2.3~2.8% (see Additional File 3, Figure S2C). These data are consistent with another report that showed the induction of CD4^+^CD25^- ^Tregs by TGF-β in murine peripheral T cells [42]. We observed higher CD25^- ^and CD25^+ ^IL-10-secreting Tr1 cells induced by VP1 (CD25^-^: 11.4%, CD25^+^: 5.9%) with cells obtained at about 1.5 years of HCV infection compared to the Tr1 cells stimulated by wild type peptide (7.6% and 2.9%) and VP2 (5.9% and 2.3%) (see Additional File 4, Figure S3). In addition, using PBMCs from another DRB1*15^+ ^patient infected with HCV over 20 years, more CD25^-^IL-10^+ ^Tr1 cells were induced by VP1 (33%) than those exposed to either wild type peptide (25%) or VP2 (17%) (see Additional File 5, Figure S4). This data s suggest that IL-10, CD25^-^TGF-β^+ ^Th3 and CD4^+^IL-10^+ ^Tr1 cells were involved in the immune inhibition by VP1, and CD25^+^TGF-β^+ ^Th3 cells played a critical role in the inhibition by VP2.

For further confirmation, we determined the levels and phenotypes of additional five HCV-infected patients who are positive for HLA-DRB1*15 (P2-P6, see Additional File 1, Table S1). The results of those patients plus patient B3019 are summarized in Table 2. Taken together, our data strongly support the hypothesis that antigenic variants of HCV are able to induce immune suppression through up-regulatory Th3, Tr1 cells and IL-10.

### Characteristics of Tregs driven by antigenic variants

**Figure 4:**
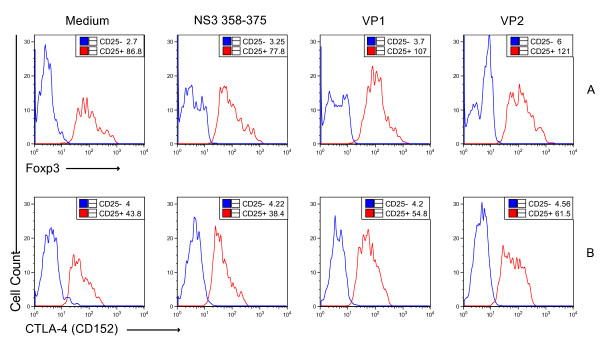
**Characteristics of antigen-driven CD25^+ ^and CD25^- ^Tregs**. Cellular culture and separation of CD4^+ ^T cells and controls were the same as described in Figure 2. The purified CD4^+ ^cells were stained with fluorescent-labeled antibodies for human CD25, CTLA-4 and *Foxp3 *followed by a flow cytometry assay. The effect of peptide NS3_358-375 _and variant pools VP1 and VP2 on expression of *Foxp3 *(**A**) and CTLA-4 (**B**) on CD4^+^CD25^- ^(blue) and CD4^+^CD25^+ ^(red) T cells is shown. Median values for each population are shown in the boxes. One representative of two experiments is shown.

The transcription factor forkhead box P3 (*Foxp3*) has been found to be a crucial regulator of immune function and is required for development of CD4^+^CD25^+ ^Tregs [43]. TGF-β has recently been reported to have an essential role in both inducing and maintaining *Foxp3 *in peripheral CD25^+ ^and CD25^- ^Tregs[44]. In addition, cytotoxic T lymphocyte-associated antigen 4 (CTLA-4, or CD152) is also expressed on CD4^+^CD25^+ ^Tregs, and CTLA-4 signaling is required for their function[45]. Therefore, we asked whether the variant-driven CD25^- ^and CD25^+ ^Tregs observed in HCV infection express CTLA-4 and *Foxp3*. The expression of CTLA-4 and *Foxp3 *in/on CD4^+^CD25^+ ^and CD4^+^CD25^- ^cells were determined by intracellular staining and flow cytometry. As shown in Figure 4, the levels of *Foxp3 *(Figure 4A) and CTLA-4 (Figure 4B) induced by exposure to VP1 and VP2 are significantly higher in CD25^+ ^cells compared to those induced by wild type peptide NS3_358-375 _(Figure 4A:107, 121 versus 77.8 for *Foxp3*; Figure 4B: 54.8, 61.5 versus 38.4 for CTLA-4). The wild type peptide NS3_358-375 _actually down-regulated the expression of *Foxp3 *and CTLA-4 compared to cells cultured with medium alone. We did not detect significant levels of CTLA-4 and *Foxp3 *expression in/on CD4^+^CD25^- ^cells. This observation suggests that: 1) the features of antigen-inducible CD4^+^CD25^+ ^Tregs in HCV infection are similar to those of natural CD4^+^CD25^+ ^Tregs although their developmental pathways are distinct; 2) the antigen-inducible CD25^- ^Tregs driven by antigenic variants are different from the CD25^- ^Tregs reported by others [44] because those CD25^- ^Tregs do not express *Foxp3*.

### *In vivo *evidence of Tr1 and Th3 cells in HCV infection

Considering the common existence of virus quasi-species and antigenic variants in chronic HCV infection [24,46], the immune evasion by antigenic variants described above is unlikely an occasional event. If that is true, we should detect elevated Tr1 and/or Th3 cells in other HCV infected patients. Direct T-subset counting by flow cytometry is a routing lab test for HIV infection and the results reflect the T cell numbers *in vivo*. Similarly, direct determination of Treg phenotypes using fresh PBMCs without *in vitro *culture would reflect Tregs induced by HCV quasi-species including antigenic variants *in vivo*. This approach can overcome or complement the shortage of *in vitro *study because it can better reflect an actual immune response *in vivo *as being used for HIV infection.

Using freshly isolated PBMCs from six other patients infected with HCV for about 2-5 years (see Additional File 1, Table S1. P7-P12) and six healthy subjects (as controls), we observed the following significant differences in HCV infected patients compared to the controls: 1) higher CD4^+^CD25^+ ^cells (17~34% versus 9~10%, p = 0.02); 2) 5~10 fold higher CD25^+ ^Th3 cells, and 15~20 fold higher CD25^- ^Th3 cells; 3) higher CD25^+^Il-10^+ ^Tr1 cells and the Tr1 fraction in two patients as high as 14.4% and 16.2%; 4) the majority of the Tr1 cells are also TGF-β^+^. In addition, patients with early HCV infection have higher serum levels of IL-10 and TGF-β (see Additional File 6, Figure S5). These results demonstrate that Th3 and Tr1 cells are up-regulated in early chronic HCV infection which is characteristic of the immune modulation induced by virus quasi-species and antigenic variants. These data are consistent with the results presented above and provide evidence to support the hypothesis that antigenic variants of HCV are able to modulate a Th1 response through regulatory Th3, Tr1 cells and IL-10.

## Discussion

We have previously reported that CD4 antigenic variants driven by immune selection clustered within immune-dominant epitopes of the HCV nonstructural three (NS3) gene [47]. Consistent to our data, a study showed HCV escapes CD8 T-cell immune response in HLA-B27+ patients through accumulated mutations within an CD8 immune-dominant epitope, viral protein RdRp which is bound by HLA-B27 molecule [28]. In the present study we provide evidence that naturally occurring CD4 mutants of HCV act as antigenic variants that transfer a host protective peripheral Th1 immune response into an inhibitory Th3 or Tr1 response through mutiple mechanisms including: 1) induction of failed T cell activation and simultaneous induction of CD4^+^CD25^- ^Th3 and CD4^+^IL-10^+ ^Tr1 cells; 2) up-regulation of CD4^+^CD25^+^Th3 cells; and 3) direct inhibition of IL-10.

The T cell receptor (TCR) exhibits an exquisite specificity for its antigen as demonstrated by the significantly different biological outcomes induced by ligands that differ by only a single amino acid [48]. VP 1 consisted of variant peptides with single or double amino acid substitutions located at the center of the core region of the Th1 peptide (see Table 1), and they can be either within or close to the TCR binding sites. Structural or conformational alteration of TCR binding sites of peptides can induce incomplete T cell activation that favors induction of anergy [49]. Indeed, our data show that VP1 peptides dramatically decreased CD4 T cell proliferation (see Figure 1A). Furthermore, CD4 cells cultured with VP1 expressed fewer T cell activation, as determined by low level expression of the T cell activation markers of CD134 and CD45RB^high ^(see Additional File 7, Figure S6), providing direct evidence of failed T cell activation by VP1 peptides. CD134 is expressed only on activated T cells, particularly activated CD4^+ ^T cells [50]. Interestingly, VP1 not only caused failed T-cell activation, but also at the same time up-regulated the regulatory cytokines IL-10 and TGF-β and either CD25^- ^Th3 or Tr1 Tregs.

The amino acid substitutions in VP2 peptides are not likely involved in TCR binding directly since they are located outside the core region of the Th1 peptide (see Table 1). However, they may act as interface-disrupting residues which can actively disrupt the TCR binding ability [51]. It is also possible that the amino acid substitutions in VP2 peptides induce conformational changes either in the TCR or in the HLA molecules on APCs. Minor changes at the TCR or APC level could have a significant impact on T cell responses, since specific T cell recognition is strictly determined [49]. In support of this hypothesis, T cells pulsed with VP2 peptides failed to respond to the subsequent challenge with the Th1 peptide NS3_358-375 _(see Figure 1). This induction of anergy was not due to incomplete T cell activation since VP2 stimulated even higher levels of the T cell activation marker CD134 than wild type peptide (see Additional File 7, Figure S6A). Because up-regulation of CD134 expression by VP2 was accompanied by the highest levels of TGF-β and CD25^+ ^Th3 Tregs, higher CD134 expression by VP2 might reflect activation of pathways related to CD25^+ ^Treg differentiation instead of T effecter activation. These results suggest that VP2 peptides also work as antigenic variants through a distinct pathway.

Collectively, the immune evasion by antigenic variants has two features. First, the modulatory effect of antigenic variants on CD4 response is effective and extensive. As low as 0.1 μM of variant peptide and as many as 17 out of 18 (94%) of the antigenic variants can induce anergy (Figure 1 and unpublished data). Multiple inhibitory components such as IL-10 as well as Th3 and Tr1 cells were induced by these variants (see Figure 2, 3). Second, induction of a deviated immune response or antigen-inducible Tregs is favored over induction of a Th1 response. It seems that a specific Th1 response requires strict TCR engagement and even single amino acid alteration of a Th1 peptide can shift the direction of a Th1 response to an inhibitory direction [48]. In contrast, induction of anergy or antigen-inducible Tregs does not require strict TCR engagement since extensive variants induced similar results of anergy and up-regulated Tregs although they may use different pathways [49]. These features provide a potential mechanism by which a minority of circulating antigenic variants can effectively suppress a protective Th1 response to native viral epitopes. Together with the feature of high mutability of HCV, this hypothesis can explain why the majority of HCV infected patients progress to persistent infection.

Consistent with the studies by others [34], our current data show an elevated ratio of IL-10^+ ^Tr1 cells in the early course (less than 5 years) of HCV infection (see Table 2). Moreover, the suppression of T cell proliferation by VP1 peptides was abrogated by anti-IL-10 antibody (see Figure 2B). We detected both CD25^- ^and CD25^+ ^Tr1 cells induced by VP1 as early as 1.5 years of HCV infection (see Additional File 3, Figure S2) whereas the majority of IL-10^+ ^cells beyond 2.5 years of infection were predominantly CD25^+^.

Regardless the cytokine sources, VP1 consistently produced higher levels of IL-10 and CD25^- ^Th3 cells, which directly contributed to the immune modulation by this group of variants. These data suggest that as antigenic variants such as VP1 evolved in the earlier course of HCV infection they initiate the regulatory mechanisms mediated by IL-10 and Th3 cells in order to escape immune pressure. Such VP1 variants up-regulate immunoregulatory IL-10 and Th3 cells that suppressed a protective Th1 response but did not influence the Th3 or Tr1 response. As a result of the existense of VP1, the balance of immune responses shifts to a Tr1 or Th3 profile as we observed in our previous [39] and present studies. This hypothesis is further supported by our data that: 1) the majority of variants as early as about one year into HCV infection consist of VP1 (see Table 1) [47]; and 2) patients infected with HCV for less than 5 years have significantly higher percentages of Tr1 and/or Th3 cells and higher serum levels of IL-10 and TGF-β (see Table 2 and Additional File 5, Figure S4).

TGF-β-secreting CD25^+ ^and CD25^- ^Th3 cells induced by antigenic variants occurred in early years of HCV infection. The immune inhibition induced by VP1 was abolished completely by deletion of TGF-β^+ ^cells rather than by blocking or deletion of CD25^+ ^cells, suggesting that this suppressive pathway by VP1 was not CD25-dependent but actually TGF-β-dependent. In contrast, the suppression induced by VP2 was CD25 and TGF-β dependent because either antibody to CD25 or deletion of CD25^+ ^and TGF-β^+ ^cells eliminated the suppressive effect. Accordingly, higher ratios of CD25^-^TGF-β^+ ^Th3 cells were observed for VP1 and VP2 (see Figure 3D) and VP2 dramatically increased the ratio of CD25^+^TGF-β^+ ^Th3 cells (see Figure 3B). In the presence of high levels of IL-2 and TGF-β produced by exposure to VP2 (see Figure 2G), CD25^-^TGF-β^+ ^cells likely differentiated to become CD25^+^TGF-β^+ ^cells [52]. The fact that deletion of TGF-β^+ ^cells instead of neutralization of TGF-β reversed the immune suppression by both VP1 and VP2 indicates that cell contact of TGF-β-bound cells instead of soluble TGF-β is necessary for the inhibitory effects. These data are consistent with the results reported by Carrier et al. Their data showed that the suppressive effect of TGF-β on T cell responses is due to the induction of Tregs instead of direct inhibition of TGF-β on T cell proliferation[44]. The finding of higher ratio of CD25^-^TGF-β^+ ^cells induced by VP1 supports the hypothesis that CD25^- ^Th3 cells are the major source of TGF-β^+ ^cells that play an important role in the immune evasion induced by VP1.

A recent study suggested that CD4 T cells function through other mechanisms rather than exerting immune selection in HCV infection [53]. Consistently, the current study indicates possible inhibitory mechanisms used by CD4 T cells in chronic HCV infection. The immune modulation can be initiated or enhanced by naturally occurring antigenic variants of a CD4 epitope. This role of CD4 cells would in turn attenuate the functional efficiency of CD8 T cells that favors HCV persistence. It should be noted that data being obtained with PBMCs collected at one time point of an infection course would only represent "snapshots" of the immune response during entire HCV infection. Additional studies would be needed to precisely correlate the occurrence of antigenic variants with the loss or shift of HCV-specific T cell responses. The phenotypes of immune responses may vary somewhat among different subjects depending on the HCV time continuum and the subjects. Samples collected at different time points from any given subject might also induce variable results. However, the overall trend of immune inhibition induced by CD4 variants during the course of HCV infection was consistent.

## Conclusions

In summary, our data revealed multiple mechanisms of peripheral immune modulation initiated or enhanced by naturally occurring antigenic variants of a CD4 epitope in the early course of HCV infection. Antigenic variants of a Th1 peptide can down-regulate a Th1 response by hampering T cell activation and simultaneous induction of IL-10, CD25^-^TGF-β^+ ^Th3 and CD4^+^IL-10^+ ^Tr1 cells. IL-10 alone can directly suppress T cell proliferation and Th1 cytokine production. In addition, other HCV variants tend to induce higher levels of TGF-β that promote differentiation of CD25^+ ^Th3 suppressors. The modulation of antigenic variants on CD4 response is efficient and extensive that is likely critical in viral persistence.

## List of abbreviations

HCV: Hepatitis C virus; CD: cluster of differentiation; NS: non-structural; PBMC: peripheral blood mononuclear cell: HIV: human immunodeficiency virus; Th: T-help; HLA: Human leukocyte antigen; DRB1: HLA class II histocompatibility antigen; DRB1beta chain; IL: Interleukin; TGF: Tumor growth factor; IFN: Interferon; Treg: T-regulatory.

## Competing interests

The authors declare that they have no competing interests.

## Authors' contributions

JHW participated in the study design, supervising experiments, data analysis and manuscript drafting. MJP, XK, and AH participated in performing the experiments, data collection and analysis. RH and SJ provided the subject samples and data collection, financial support. WC, ZC, and LR participated in data analysis and manuscript drafting. All authors have read and approved the final version of the manuscript.

## Supplementary Material

Additional file 1 **Table S1**. The information of HCV-infected patients. The information of HCV-infected patients. Click here for file

Additional file 2 **Figure S1**. An example of CD4+CD25+ cells achieved by culturing HCV-infected PBMCs with variant peptide pool VP1 at presence (B) or absence (A) of anti-TGF-βantibodies; and CD4+CD25+ cells achieved by culturing HCV-infected PBMCs with (D) or without (C) TGF-β^+^cell deletion. Either anti-TGF-β antibodies or deletion of TGF-β^+^cells significantly reduced CD4+CD25+ cells. Two representatives of four experiments are shown. Click here for file

Additional file 3 **Figure S2**. CD4+CD25+ and CD4+CD25- Tregs of a HCV- healthy individual. Experiments, controls, and data analysis were the same as described in Figure 4. A: Percentages of CD25+ and CD25- gated CD4+ cells. B: Percentages of CD25+ Th3 and Tr1 cells. C: Percentages of CD25- Th3 and Tr1 cells. A single representative of three experiments is shown. Click here for file

Additional file 4 **Figure S3**. Treg phenotypes induced by peptide NS3_358-375 _and variant pools VP1 and VP2 at about 1.5 years post HCV infection. Cell culture and purification of CD4^+ ^cells and controls were the same as described in Figure 4. Intracellular staining of purified CD4+ cells was performed using fluorescent-labeled antibodies to human CD25, IFN-γ, TGF-β and IL-10. **A**: Percentages of CD25^-^IL-10^+ ^Tr1 cells. **B**: Percentages of CD25^+^IL-10^+ ^Tr1 cells. A single representative of three experiments is shown. Click here for file

Additional file 5 **Figure S4**. Treg phenotypes from a patient infected with HCV for over 20 years. Cell culture, purification of CD4^+ ^cells, controls, intracellular staining and flow cytometry analysis were the same as described in Figure 2. Note that VP1 peptides induced higher CD25^-^Il-10^+ ^Tr1 cells than wild type peptide NS3_358-375_, VP2 peptides and medium alone. Click here for file

Additional file 6 **Figure S5**. Serum levels of IL-10 and TGF-β. C: HCV- healthy controls; P: HCV+ patients. Click here for file

Additional file 7 **Figure S6**. HCV variants induced incomplete T cell activation and/or up-regulated CD45RB^low ^Tregs. PBMCs were cultured with peptide NS3_358-375 _(5 μM) and variant pools (1 μM/each peptide) for 66 hours followed by positive selection of CD4^+ ^T cells with anti-human CD4 antibodies and magnetic beads. The CD4^+ ^cells obtained were then stained with fluorescent-labeled antibodies to human CD134 (OX40), and CD45RB and analyzed using a three-color flow cytometer. PBMCs cultured with medium alone were used as control. The effect of peptide NS3_358-375 _and variant pools VP1 and VP2 on expression of CD134 (**A**) and CD45RB (**B**) on CD4 T cells is demonstrated. A representative of two experiments is shown. Click here for file
