# Supplementary material for: Characterization of antigenic variants of hepatitis C virus in immune evasion
Source: Virol J. 2011 Jul 29;8:377. doi: 10.1186/1743-422X-8-377 (PMC3158126; doi:10.1186/1743-422X-8-377)
Supplement: Supplementary file 1 [file 1743-422X-8-377-S1.DOC]

Table S1. The information of HCV-infected patients

ID	Age	Genda	Race	Year of infection	HCV genotype	HLA-DRB1*
P1	33	F	C*	4.0	1B	07,15
P2	23	M	C	4.5	1A	07,15
P3	21	M	C	2.7	1	04,15
P4	24	M	C	4.3	3A	04,15
P5	25	F	C	4.0	2A	12,15
P6	22	M	C	4.2	1A	03,15
P7	28	F	C	3.0	1	01,13
P8	30	M	C	3.4	1B	01,03
P9	30	M	C	3.4	1A	03,07
P10	21	M	C	4.1	1A	04,13
P11	23	F	C	1.5	1A	07,11
P12	28	F	HL**	2.3	1A	11,15

*: Caucasion. **: Hispanic Latino
