# Supplementary material for: Characterization of antigenic variants of hepatitis C virus in immune evasion
Source: Virol J. 2011 Jul 29;8:377. doi: 10.1186/1743-422X-8-377 (PMC3158126; doi:10.1186/1743-422X-8-377)
Supplement: Supplementary file 2 [file 1743-422X-8-377-S2.DOC]

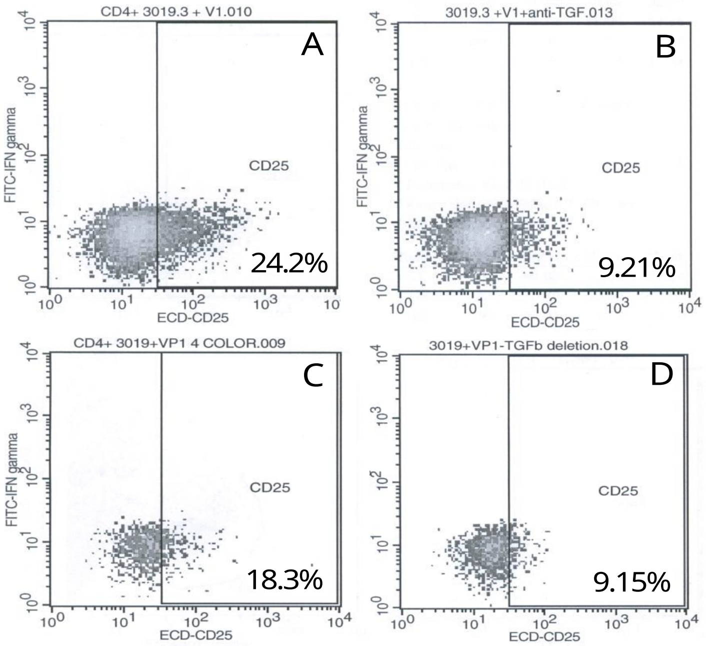

CD4+ 3019.3 + V1.010
A
CD25
24.2%
3019.3 +V1+anti-TGF.013
B
CD25
9.21%
CD4+ 3019+VP1 4 COLOR.009
C
CD25
18.3%
3019+VP1-TGFb deletion.018
D
CD25
9.15%
FITC-IFN gamma
ECD-CD25
