# Supplementary material for: Characterization of antigenic variants of hepatitis C virus in immune evasion
Source: Virol J. 2011 Jul 29;8:377. doi: 10.1186/1743-422X-8-377 (PMC3158126; doi:10.1186/1743-422X-8-377)
Supplement: Supplementary file 3 [file 1743-422X-8-377-S3.DOC]

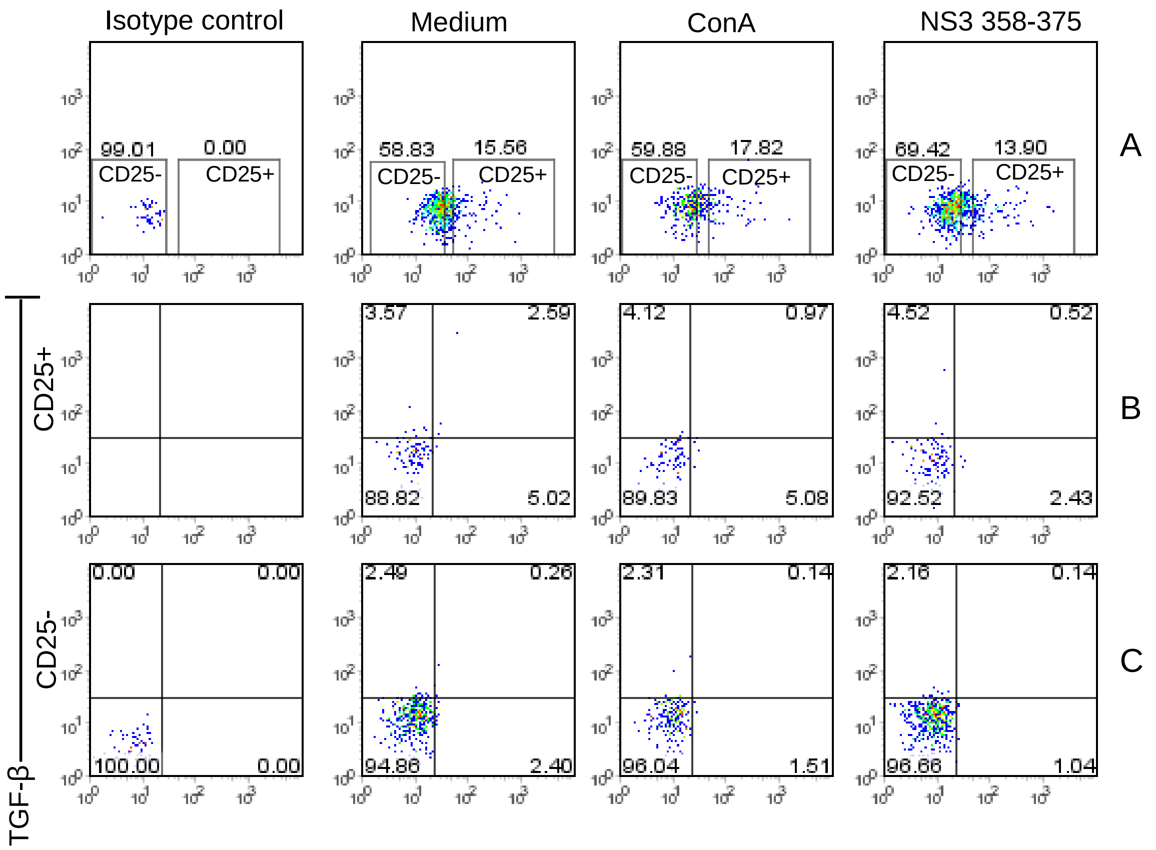
Isotype control
Medium
ConA
NS3 358-375
99.01
0.00
CD25-
CD25+
58.83
15.56
59.88
17.82
69.42
13.90
A
B
C
CD25+
CD25-
TGF-β
3.57
2.59
88.82
5.02
4.12
0.97
89.83
5.08
4.52
0.52
92.52
2.43
0.00
0.00
100.00
0.00
2.49
0.26
94.86
2.40
2.31
0.14
96.04
1.51
2.16
0.14
96.66
1.04
