# Supplementary material for: Characterization of antigenic variants of hepatitis C virus in immune evasion
Source: Virol J. 2011 Jul 29;8:377. doi: 10.1186/1743-422X-8-377 (PMC3158126; doi:10.1186/1743-422X-8-377)
Supplement: Supplementary file 4 [file 1743-422X-8-377-S4.DOC]

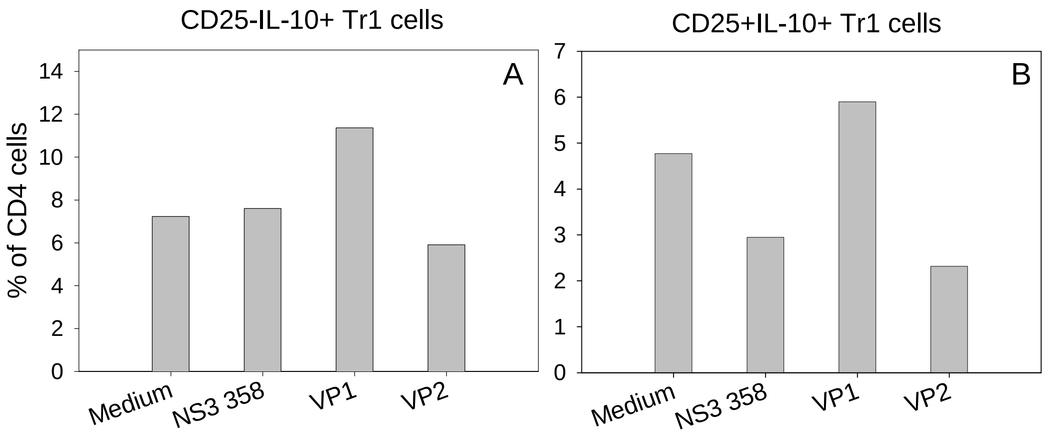
CD25-IL-10+ Tr1 cells
A
% of CD4 cells
0
2
4
6
8
10
12
14
Medium
NS3 358
VP1
VP2
CD25+IL-10+ Tr1 cells
B
0
1
2
3
4
5
6
7
Medium
NS3 358
VP1
VP2
