# Supplementary material for: Characterization of antigenic variants of hepatitis C virus in immune evasion
Source: Virol J. 2011 Jul 29;8:377. doi: 10.1186/1743-422X-8-377 (PMC3158126; doi:10.1186/1743-422X-8-377)
Supplement: Supplementary file 5 [file 1743-422X-8-377-S5.DOC]

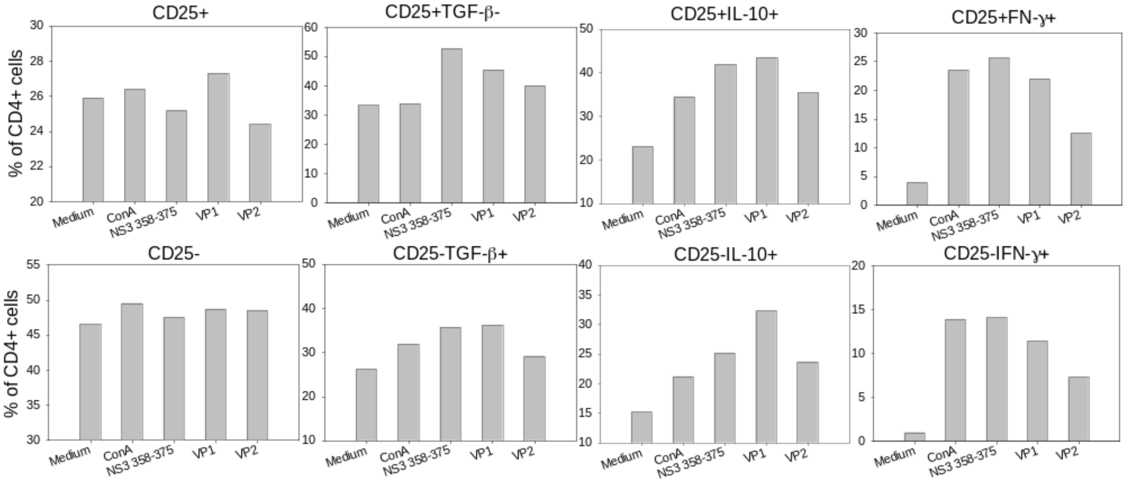
CD25+
CD25+TGF-β-
CD25+IL-10+
CD25+FN-γ+
CD25-
CD25-TGF-β+
CD25-IL-10+
CD25-IFN-γ+
% of CD4+ cells
Medium
ConA
NS3 358-375
VP1
VP2
