# Supplementary material for: Characterization of antigenic variants of hepatitis C virus in immune evasion
Source: Virol J. 2011 Jul 29;8:377. doi: 10.1186/1743-422X-8-377 (PMC3158126; doi:10.1186/1743-422X-8-377)
Supplement: Supplementary file 6 [file 1743-422X-8-377-S6.DOC]

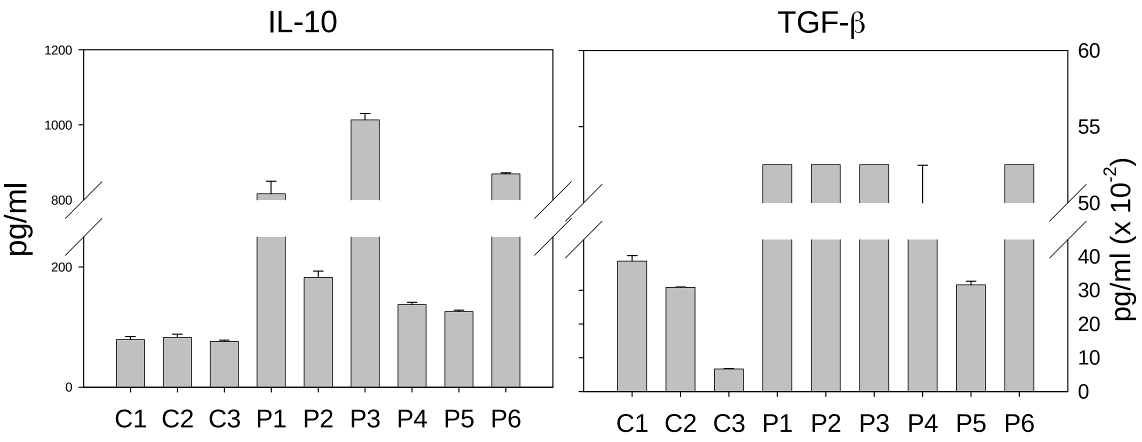
IL-10
TGF-β
pg/ml
pg/ml (x 10⁻²)
1200
1000
800
200
0
60
55
50
40
30
20
10
0
C1 C2 C3 P1 P2 P3 P4 P5 P6
C1 C2 C3 P1 P2 P3 P4 P5 P6
