# Supplementary material for: Characterization of antigenic variants of hepatitis C virus in immune evasion
Source: Virol J. 2011 Jul 29;8:377. doi: 10.1186/1743-422X-8-377 (PMC3158126; doi:10.1186/1743-422X-8-377)
Supplement: Supplementary file 7 [file 1743-422X-8-377-S7.DOC]

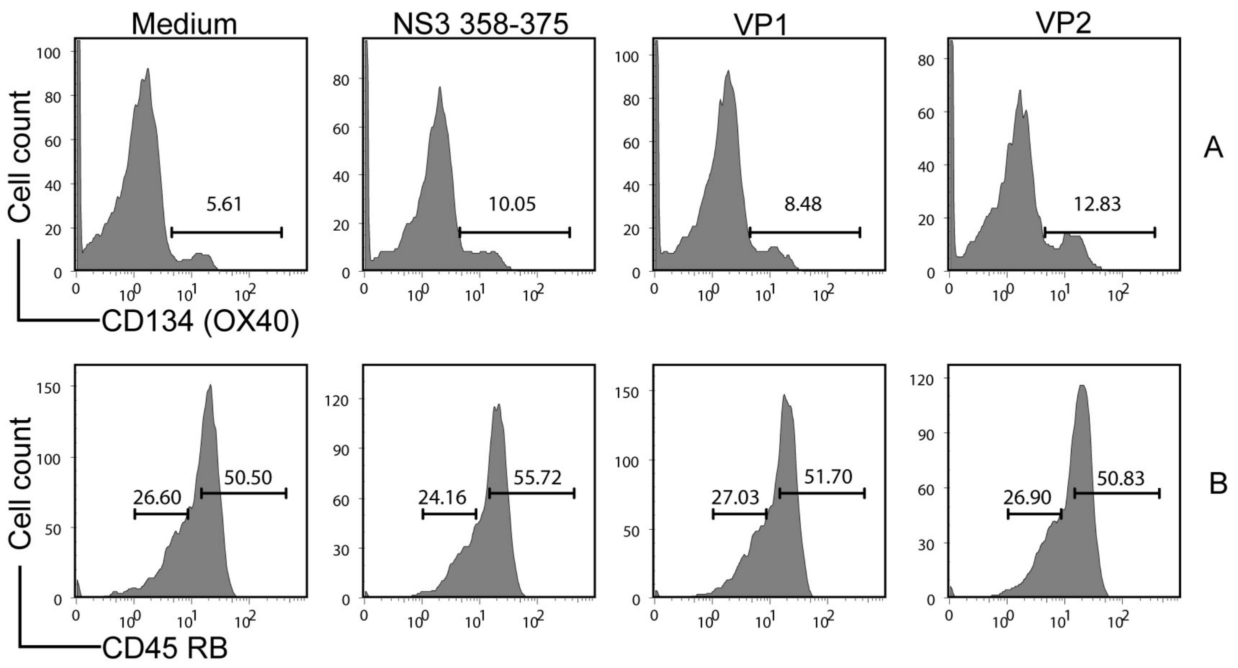

Medium
NS3 358-375
VP1
VP2
Cell count
CD134 (OX40)
5.61
10.05
8.48
12.83
A
Cell count
CD45 RB
26.60
50.50
24.16
55.72
27.03
51.70
26.90
50.83
B
